# Uveitis–glaucoma–edema (UGE): a clinical triad in un-treated helminth-associated severe anterior segment inflammation

**DOI:** 10.1186/s12348-025-00531-4

**Published:** 2025-11-05

**Authors:** Rakesh Shakya, Gaurav Kohli, Chintan Shah, Ananya Sinha, Gautam Parmar, Navjot Singh Ahluwalia, Deepak Edward, Alok Sen

**Affiliations:** 1 Department of Glaucoma, Sadguru Netra Chikitsalaya, Jankikund, Chitrakoot, Satna, Madhya Pradesh India; 2 Vitreo-Retina, Uvea, and ROP- Department of Ophthalmology, Eras Medical College and Hospitals, Lucknow, India; 3 Department of Pediatric ophthalmology and strabismus, Sadguru Netra Chikitsalaya, Madhya Pradesh Chitrakoot, India; 4 Department of Cornea and Refractive Surgery, Sadguru Netra Chikitsalaya, Chitrakoot, India; 5 Department of Ophthalmology, Illinois Eye and Ear Infirmary, University of Illinois, Chicago, IL USA; 6 Department of Retina and Uvea, Sadguru Netra Chikitsalaya, Madhya Pradesh Chitrakoot, India

**Keywords:** Ocular parasitosis, Parasitic uveitis, Parasite in eye, Ocular helminthic infection, UGE syndrome

## Abstract

**Purpose:**

To describe the clinical presentation, treatment response, and surgical management of anterior segment ocular helminthic infections.

**Methods:**

This single-center case series included patients diagnosed with helminthic anterior uveitis based on the presence of a characteristic triad: anterior uveitis, elevated intraocular pressure (IOP), and corneal edema. All patients were initially treated with topical corticosteroids and antiglaucoma medications. Definitive treatment consisted of surgical extraction of the intraocular helminth after adequate control of inflammation and IOP.

**Results:**

All cases presented with Uveitis, raised IOP (Glaucoma), and corneal Edema (UGE) triad, which significantly impacted initial visualization and diagnosis. A non-touch surgical technique using viscoelastic-assisted dissection and capsulorhexis forceps was employed to extract the intraocular worms. Microscopic identification confirmed the organisms as *Gnathostoma spinigerum*, *Wuchereria bancrofti*, and *Loa loa*.

**Conclusion:**

Helminthic infestation of the anterior segment, although rare, may present as a distinct clinical entity characterized by uveitis, secondary glaucoma, and corneal edema. Early recognition of this triad can raise clinical suspicion. Controlled inflammation and IOP normalization, followed by non-traumatic surgical extraction, are key to favorable outcomes.

## Introduction

Ocular parasitosis represents a rare and often under-recognized cause of infectious uveitis. Clinically, they may present as non-specific orbital inflammation, severe intraocular inflammation with painful vision loss, secondary inflammatory glaucoma, or with delayed diagnosis due to low suspicion, resulting in vision-threatening outcomes. [1] It is infrequently encountered in clinical practice, frequently misdiagnosed, and seldom considered in the differential diagnosis of anterior uveitis. Parasitic infections tend to be more prevalent in individuals from lower socioeconomic backgrounds, particularly in tropical regions where poor sanitation and hygiene conditions are common [2–4]. 

Parasitic uveitis is broadly classified into protozoal, helminthic, and ectoparasitic. Among these, helminthic infections pose unique diagnostic challenges, mimicking other ocular inflammations and following atypical courses, like viral trabeculitis and fuchs heterochromic uveitis- these can mimic inflammatory glaucoma.

Intraocular helminthiasis is exceedingly rare and represents a severe systemic parasitic disease. Nematodes, cestodes, and trematodes can all be implicated, frequently presenting as visceral larva migrans. Diagnosis is complicated by non-specific signs, poor visibility of the parasite, and often routine systemic workup, leading to uncertainty and delayed treatment [5, 6]. 

The literature on parasitic uveitis remains scarce, primarily because it is an atypical and evolving condition. Most available information is limited to isolated case reports and small case series, which highlight the wide spectrum of clinical presentations. This report describes three cases of ocular helminthiasis with a consistent triad of anterior uveitis, glaucoma, and corneal edema. We suggest that this clinical constellation may offer a valuable diagnostic clue, raising suspicion of helminthic origin and aiding timely intervention.

### Case 1

**Fig. 1:**
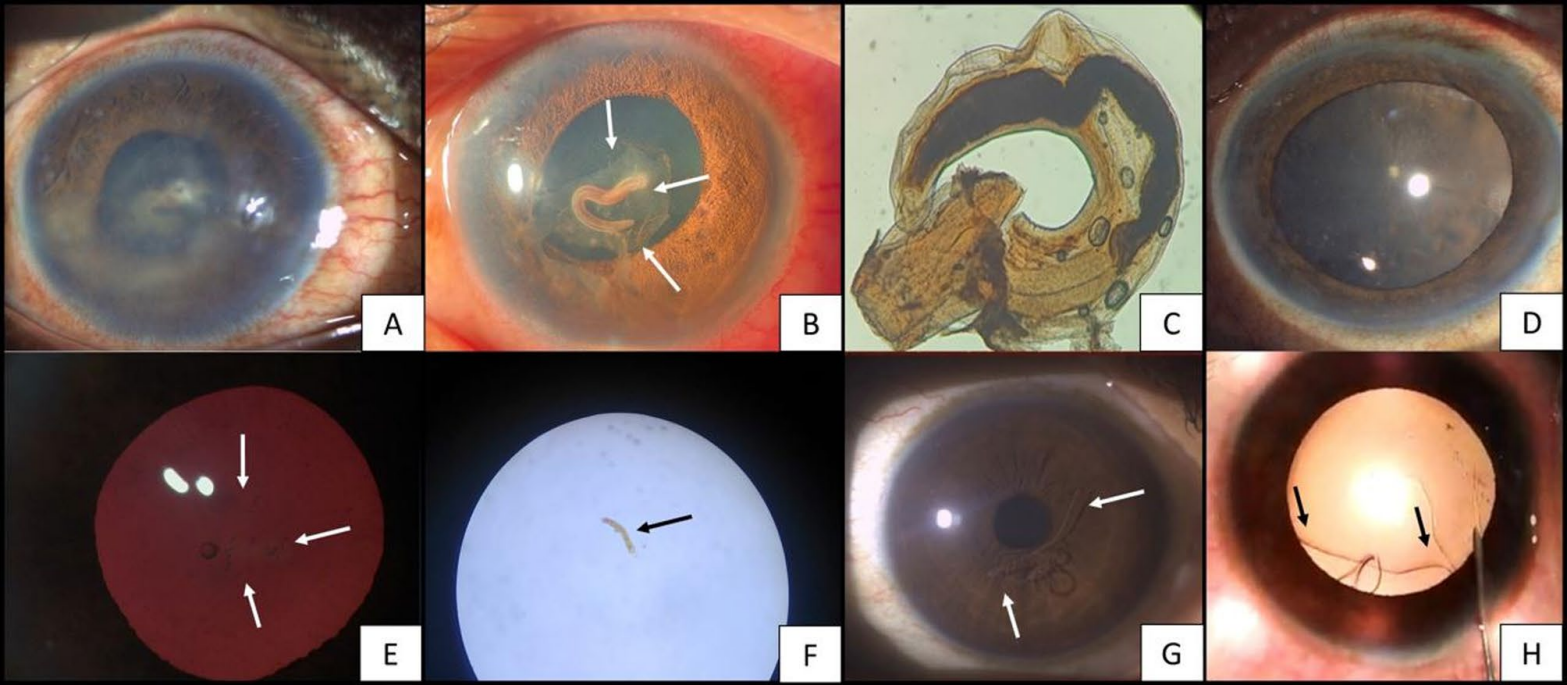
White and black arrows indicate the presence of a worm. (**A**) Case 1- slit lamp photograph showing corneal edema, hazy anterior chamber along with fibrinous membrane over anterior lens capsule, (**B**) Case 1- slit lamp photo five days after starting medical treatment. The image shows a clear cornea, a persistent fibrin membrane, engulfing a cylindrical worm, atop the anterior lens capsule (**C**) Case 1- Microscopic analysis revealing a third-stage larva of *Gnathostoma spinigerum*, (**D**) Case 1- One month follow-up after removing the worm showing clear cornea and quiet anterior chamber. (**E**) Case 2- thread-like worm attached to anterior lens capsule, with presence of corneal haze due to edema (**F**) Case 2- Microscopic examination of the larvae reveals immature stages of *Wuchereria bancrofti*. (**G**) Case 3- large thread-like worm in the anterior chamber, (**H**) Case 3- viscoelastic-assisted compartmentalization followed by careful aspiration of the worm

A 35-year-old male presented with an acute onset of painful diminution of vision in the right eye for one week. The best-corrected visual acuity (BCVA) was 6/36 in the right eye and 6/6 in the left eye. Slit-lamp examination of the right eye revealed ciliary congestion and corneal edema. The anterior chamber reaction was too dense to be graded; however, a well-organized fibrinous membrane over the anterior lens capsule was visible. [Figure 1A] Intraocular pressure (IOP), measured using Goldmann applanation tonometry, was elevated at 30 mmHg in the right eye, compared to 14 mmHg in the left.

Systemic and topical antiglaucoma therapy, corticosteroids and cycloplegics were given. After a week of treatment, corneal edema subsided and IOP normalized, allowing for improved visualization of anterior segment structures. On re-evaluation, multiple iris atrophic patches and posterior synechiae were noted. A dense, plaque-like fibrin membrane persisted on the anterior lens capsule, within which a cylindrical, fleshy, salmon-pink, non-motile worm was seen embedded. [Figure 1B]

Surgical removal of the worm was planned via a trans-limbal approach under peribulbar anesthesia. A Capsulorhexis forceps was used to lift the fibrin membrane off the anterior lens capsule along with the enmeshed worm, following which it was delivered via viscoelastic expression. Gross examination revealed a stout, coiled, brownish-salmon pink larva, approximately 0.8–1.0 cm in length. Microscopic analysis confirmed the diagnosis of *Gnathostoma spinigerum*. [Figure 1C] A wet mount revealed a third-stage larva with a distinct bulbous head bearing four rows of hooklets, and a body lined with transverse rows of cuticular spines. One month later, the patient had clear cornea, quiet anterior chamber, and minimal anterior subcapsular cataract formation and an unremarkable posterior segment. [Figure 1D]

### Case 2

An 83-year-old female presented with an acute onset of painful diminution of vision in the left eye for three days. BCVA at presentation was 6/6 in the right eye and light perception in the left eye. Examination of left eye revealed diffuse microcystic corneal edema and an elevated IOP of 44 mmHg.

The patient was started on oral and topical antiglaucoma medications, corticosteroids and cycloplegics. As the IOP lowered and the corneal edema cleared, anterior segment evaluation revealed low-grade anterior uveitis and multiple slender, thread-like worms within the anterior chamber, some freely suspended and others adherent to anterior lens capsule. [Figure 1E] These worms appeared translucent and actively exhibited wriggling movements in response to light. Gonioscopy revealed a clustering of worms in the anterior chamber angle, with a higher density in the inferior quadrant.

Fundus examination showed no signs of inflammation. However, the optic nerve head revealed an increased cup-to-disc ratio of 0.9, with near-total neuroretinal rim loss and pronounced rim pallor, suggesting glaucomatous optic neuropathy. No parasites were observed in the vitreous cavity.

Systemic examination was unremarkable—no subcutaneous nodules or migratory skin tracks were identified. Hematologic evaluation revealed peripheral eosinophilia and mild anemia, while other routine parameters, including ESR (25 mm/Hour), were within normal limits.

The patient underwent surgical removal of the intraocular parasites. Aqueous fluid and anterior capsule specimens were sent for microbiological analysis. Microscopic examination identified the larvae as immature stages of *Wuchereria bancrofti*. [Figure 1F]

### Case 3

A 32-year-old female presented with complaints of ocular discomfort and blurring of vision in the right eye for 5 days. The vision in the affected eye was reduced to 6/24 at presentation. Clinical examination revealed corneal edema and an elevated IOP of 30 mmHg. Slit-lamp evaluation demonstrated a hypermotile, translucent, thread-like worm within the anterior chamber, accompanied by a grade 1 cellular reaction. [Figure 1G]

The patient was scheduled for viscoelastic-assisted compartmentalization followed by careful aspiration of the worm. [Figure 1H] Following the procedure, there was a notable reduction in both IOP and intraocular inflammation. Pathological examination of the extracted parasite confirmed it to be an adult female *Loa loa*. The visual acuity returned to normal (6/6) over 2 weeks.

In all three cases, considering the absence of definitive systemic manifestations, long-term antihelminthic therapy was not initiated. Instead, a single prophylactic dose of albendazole 400 mg was administered following a thorough evaluation by a physician.

## Discussion

In this case series of ocular helminthiasis involving the anterior segment, we observed a consistent and clinically significant triad—anterior uveitis, glaucoma, and corneal edema (UGE)—across all three cases. This triad developed specifically when the parasite was located in the anterior chamber, and it played a key role in both symptomatology and diagnostic suspicion. Corneal edema initially obscured visualization of the intraocular worm, but their resolution following appropriate medical management allowed for identification and subsequent surgical extraction. The causative organisms were confirmed to be *Gnathostoma spinigerum*, *Wuchereria bancrofti*, and *Loa loa*, respectively.

Topical antiglaucoma medications were initiated prior to surgery in all three cases to manage elevated IOP. Following surgery, topical corticosteroids were prescribed and tapered weekly, depending on the degree of intraocular inflammation. Antiglaucoma medications were continued for one week postoperatively, after which they were discontinued, except in case 1, who showed advanced disc cupping and nerve fiber loss. In all cases, the IOP normalized and remained stable without medication after the first week of surgery.

Due to the migratory nature and rarity of intraocular nematode infections, the precise route of ocular entry remains uncertain. However, indirect clinical evidence—such as early-stage anterior segment inflammation in the absence of posterior segment involvement—suggests that primary invasion of the anterior chamber may occur in certain cases.

A review of the literature on ocular Gnathostomiasis identified 13 published reports, of which six described iris perforations, with inflammation predominantly localized to the anterior segment; these cases also had significant posterior segment involvement. These findings suggest that iris defects may serve as potential pathways for worm migration and may precede posterior segment involvement [7]. 

Biswas et al. described iris defects in cases where *Gnathostoma* had migrated into the posterior segment via the iris diaphragm [8, 9]. In contrast, in our series, only one patient exhibited iris atrophy without frank iris defects, and all parasites were confined to the anterior chamber. This raises the possibility that the absence of iris defects may suggest anterior segment confinement of the helminth. However, iris atrophy or defects are not obligatory findings and should not be considered essential for diagnosis and the route of entry could still be through the iris vasculature [10]. 

The clinical presentation of ocular helminthic infestations is highly variable, ranging from asymptomatic findings to isolated anterior uveitis, depending on the host’s immune response and the parasite’s behavior [11]. This variability contributes to frequent diagnostic delays. In our series, elevated IOP may be attributed to trabecular meshwork obstruction by inflammatory debris and parasitic presence, while the corneal edema likely resulted from sustained IOP elevation and possible inflammatory endotheliitis. Both resolved with medical therapy, suggesting an inflammatory pathogenesis rather than structural damage.

The UGE triad remained consistent in all three cases, regardless of the helminth subtype. While anterior uveitis and iris atrophy have been frequently reported in previous literature on ocular helminthiasis, our series highlights the additional components of glaucoma and corneal edema as important, and perhaps under-recognized, clinical consequences of parasitic inflammation. The elevated IOP was likely attributed to inflammatory dysfunction of the outflow pathways and the corneal edema, a combination of corneal endothelial compromise from anterior segment inflammation, elevated IOP, and presumptive prior contact by the paracyte. These findings may represent a disease continuum in neglected cases.

Observations like our study—namely, the occurrence of uveitis alongside corneal edema and/or elevated IOP have been reported by Mitra et al., Sujata et al., and Bastola et al. in association with *Wuchereria bancrofti*, *Gnathostoma*, and ocular cysticercosis, respectively [12–14]. While components of the UGE triad have been independently documented in these infections, they have seldom been emphasized as a cohesive clinical pattern. We propose that recognizing this constellation of findings may serve as a useful diagnostic cue, prompting consideration of a helminthic etiology in atypical or refractory cases of anterior uveitis.

The triad of corneal edema, uveitis, and elevated intraocular pressure is classically associated with conditions such as viral trabeculitis (particularly herpetic etiologies) and Fuchs heterochromic uveitis. However, in endemic regions, a similar constellation of findings may also arise from parasitic infestation of the anterior segment, as demonstrated in our series.

Given the geographic overlap between rural, agrarian communities and environments conducive to helminth exposure, clinicians must maintain a high index of suspicion for ocular helminthiasis when encountering hypertensive uveitis in such settings. [15]

In conclusion, clinicians should be aware of the esoteric and protean nature of intraocular helminthic infections. Our intent is not to overstate the significance of the UGE triad but to provide a fresh perspective on its diagnostic value and to recognize this triad as a possible clinical signature in patients presenting with unexplained anterior uveitis, secondary glaucoma, and corneal edema.

## Data Availability

This is a brief report containing three subjects. All necessary details have been provided within the manuscript only. There is no separate data sheet.

## Abbreviations

BCVA: Best-corrected visual acuity
IOP: Intraocular pressure
ESR: Erythrocyte sedimentation rate
UGE: Uveitis, glaucoma, and corneal edema
